# Data, Signal and Image Processing and Applications in Sensors

**DOI:** 10.3390/s21103323

**Published:** 2021-05-11

**Authors:** Manuel J. C. S. Reis

**Affiliations:** Engineering Department, University of Trás-os-Montes e Alto Douro (UTAD)/IEETA, 5000-801 Vila Real, Portugal; mcabral@utad.pt

With the rapid advance of sensor technology, a vast and ever-growing amount of data in various domains and modalities are readily available. However, presenting raw signal data collected directly from sensors is sometimes inappropriate, due to the presence of, for example, noise or distortion, among others. In order to obtain relevant and insightful metrics from the sensors signals’ data, further enhancement of the acquired sensor signals, such as the noise reduction in the one-dimensional electroencephalographic (EEG) signals or color correction in the endoscopic images, and their analysis by computer-based medical systems, is needed. The processing of the data and the consequent extraction of useful information are also vital and included in the topics of this Special Issue.

This Special Issue of *Sensors* aims to highlight advances in the development, testing, and application of data-, signal-, and image-processing algorithms and techniques to all types of sensors and sensing methodologies. Experimental and theoretical results, as well as review papers, were also considered.

The number of submitted manuscripts directly reflects the huge interest in this topic from the research community: a total of 58 manuscripts have been submitted, and 28 high-quality papers were published. As usual, the *Sensors* journal standards guided all the submitted manuscripts through a rigorous peer-review process.

In the following presentation, I will use the authors’ own words to better present the contributions of each paper.

The demand for high-quality immersive content is a primary concern for production companies and consumers, with this being pushed by the recent advancements in virtual reality (VR) and augmented reality (AR). Additionally, the topical record-breaking performance of deep learning in various domains of artificial intelligence has extended the attention of researchers to contribute to different fields of computer vision. Several learning-based Stitched Image Quality Assessment methods have been proposed with reasonable performances, trying to ensure the quality of immersive media contents using these advanced deep-learning technologies. The approximation proposed by Ullah et al. [1] can efficiently segment and localize stitching errors and estimate the image quality by investigating segmented regions. The proposed system outperformed the existing state-of-the-art techniques when extensive qualitative and quantitative comparison with full reference image-quality assessment (FR-IQA) and no reference image-quality assessment (NR-IQA) on two publicly available datasets were performed by these researchers.

In [2], the problem of remote control devices commonly used for interaction with multimedia equipment and applications (smart TVs, gaming, etc.) was addressed. To improve conventional keypad-based technologies, haptic feedback and user input capabilities are being developed to enhance the UX and provide advanced functionalities in remote control devices. As stated in the authors own words, “this work is a contribution towards UX evaluation of remote control devices with haptic feedback and text input. The user preferences, given by subjective evaluation scores, demonstrate that haptic feedback undoubtedly has a positive impact on the UX”, and they also show that “different levels of UX are obtained, according to the technological characteristics of the haptic actuators and how many of them are used on the device”.

Anvarjon, Mustaqeem and Kwon [3] have proposed a new, lightweight, effective Speech Emotion Recognition (SER) model, with a low computational complexity and a high recognition accuracy. The model uses an approach based on Convolutional Neural Networks (CNN) to learn the deep frequency features by using a plain rectangular filter with a modified pooling strategy. The proposed CNN model was trained on the extracted frequency features from the speech data and was then tested to predict the emotions. The proposed SER model was evaluated over two benchmarks, which included the interactive emotional dyadic motion capture (IEMOCAP) and the berlin emotional speech database (EMO-DB) speech datasets, and it obtained 77.01% and 92.02% recognition results, showing a better recognition performance than the state-of-the-art SER systems.

Hu and Feng [4] presented a linear dimensionality reduction method—minimum eigenvector collaborative representation discriminant projection—to address high-dimensional feature extraction problems. They used the eigenvector corresponding to the smallest non-zero eigenvalue of the sample covariance matrix to reduce the error of collaborative representation, and maintain the collaborative representation relationship of the samples in the projection subspace to enhance the discriminability of the extracted features. Additionally, the between-class scatter of the reconstructed samples was used to improve the robustness of the projection space. The experimental results (on the COIL-20 image object database, ORL, and FERET face databases, as well as Isolet database), demonstrate the effectiveness of the proposed method, especially in low dimensions and small training sample sizes.

Yuan et al. [5] proposed a demodulation method based on Loran-C Pulse Envelope Correlation–Phase Detection (EC–PD), in which EC has two implementation schemes, namely, moving average-cross correlation and matched correlation, to reduce the effects of noise and SkyWave Interference (SWI). The presented simulation results show that, compared with an existing method, the proposed method has clear advantages, and the test results show that the average data availability of the proposed method is 3.3 times higher than that of the existing methods.

Zhang et al. [6] proposed a super-resolution algorithm based on the multi-modality super-CMOS sensor which can adapt to the limited operation capacity of microsatellite computers. The results of the experiments showed that the satellite-using super-resolution algorithm, combined with multi-modality super-CMOS sensor oblique-mode sampling, can increase the spatial resolution of an image by about two times. The algorithm is simple and highly efficient, and can realize the super-resolution reconstruction of two remote-sensing images within 0.713 s.

In the paper by Karageorgos et al. [7], two novel and practical regularizing methods were proposed to improve existing neural network architectures for monocular optical flow estimation. These methods aim to alleviate the deficiencies of current methods, such as flow leakage across objects and motion consistency within rigid objects, by exploiting contextual information. The first method is architecture-agnostic and can be integrated into any neural network without modifying or adding complexity at inference. The second regularization method adds spatial awareness to the input data of the network, in order to improve the training stability and efficiency. The combination of both regularization methods further improves the performance of existing cutting edge architectures in a complementary way, both quantitatively and qualitatively, on popular flow estimation benchmark datasets.

Zhou, Ding and Zhang [8] have designed an effective end-to-end, deep-learning-based, non-blind image-deblurring algorithm. The experimental results, on the public GoPro dataset and the realistic and dynamic scenes (REDS) dataset, show that the proposed method generally outperforms some traditional deburring methods and deep-learning-based, state-of-the-art deblurring methods, such as scale-recurrent network (SRN) and denoising prior driven deep neural network (DPDNN), in terms of such quantitative indexes as peak signal-to-noise ratio (PSNR) and structural similarity (SSIM) and human vision.

Automatic segmentation of HippoCampus (HC) structures is challenging due to their small volume, complex shape, low contrast and discontinuous boundaries. Liu and Yan [9] proposed a semi-automatic model that combines a Deep Belief Network (DBN) and the Lattice Boltzmann (LB) method for the segmentation of HippoCampus (HC). The segmentation results have good correlation and consistency with the manual segmentation results.

Sumali et al. [10] presented a study inspecting the distribution and statistical characteristics of dementia patient and depression patient, and compared them. It was found that some acoustic features were shared in both dementia and depression, although their correlation was reversed. Statistical significance was also found when comparing the features. The possibility of utilizing machine learning for automatic pseudo-dementia screening was also explored. These results showed that dementia and depression might be both detected and differentiated based on acoustic features alone. Automated screening is also possible based on the high accuracy of machine learning results.

Pinto et al. [11] established a physiological model of emotions based on the processing of electrocardiogram, electromyogram, and electro-dermal activity. Unimodal and multimodal approaches were used to analyze what signal, or combination of signals, may better describe an emotional response, using a sample of 55 healthy subjects. Results suggest that the electrocardiogram (ECG) signal is the most effective for emotion classification, but the combination of all signals provides the best emotion identification performance, with all signals providing crucial information for the system.

Chen et al. [12] proposed a weak signal enhancement method based on neural-network-assisted empirical mode decomposition (EMDNN), in order to enhance weak signals in strong noise background. This method combines complementary ensemble empirical mode decomposition, generative adversarial networks and long short-term memory, enhances the efficiency of selecting effective natural mode components in empirical mode decomposition, and improves SNR. The experimental results show that the SNR of this method is improved from 4.1 to 6.2, and the weak signal is clearly recovered.

Feng and Feng [13] proposed a robust method to accurately determine the correspondences by fusing two complementary structural features, including the spatial location of a point and the local structure around it. Experimental results demonstrate that the method can achieve a better performance than several existing state-of-the-art methods.

Hu and Liu [14] proposed an $l_{m}$-norm maximization model to solve dual-principal component pursuit (DPCP), based on the similarities between DPCP and Sparse dictionary learning, and a smooth unconstrained exact penalty model, and show its equivalence with the $l_{m}$-norm maximization model. The results illustrate the high efficiency of the penalty model when compared with the other state-of-the-art algorithms in the solution of the $l_{m}$-norm maximization with orthogonality constraints.

Zhao et al. [15] proposed a preference analysis method based on residual histograms to study the outlier consensus for outlier detection. The experimental results show that the outlier detection scheme based on residual histogram preference can detect most of the outliers in the datasets, and the fitting results are better than most of the state-of-the-art methods in geometric multi-model fitting.

Radlak, Malinski and Smolka [16] presented a switching filtering technique intended for impulsive noise removal using deep learning. The results show that the proposed approach is superior to the state-of-the-art filters designed for impulsive noise removal in color digital images.

Yan et al. [17] proposed an envelope delay correlation acquisition method that, when combined with linear digital averaging technology, can effectively suppress noise and cross-rate interference. These test results show that the method can reliably detect Loran-C pulse group signals over distances up to 1500 km, even at a low signal-to-noise ratio.

Ni et al. [18] proposed a support vector domain description classifier with the particle swarm optimization algorithm for foreign object debris detection. The results, based on measured data, showed that the proposed methods could not only achieve a good detection performance but also significantly reduce the false alarm rate.

Jin, Zun and Yong [19] proposed a novel model to obtain depth images, characterised by both a low-rank structure and nonlocal self-similarity. The results show that the proposed algorithm attains state-of-the-art performance.

Liang et al. [20] propose a highly efficient optimal estimation algorithm for MEMS arrays based on Wavelet Compressive Fusion (WCF), to improve the performance of MEMS inertial devices. The experimental results demonstrate that the WCF algorithm has outstanding real-time performance and can effectively improve the accuracy of low-cost MEMS inertial devices.

Xing Wei and Guangjun Zhang [21] presented a line-matching method based on multiple intensity ordering with uniformly spaced sampling. The performance of the proposed method was tested on public datasets. The experimental results show that the method achieves a superior performance in dealing with various image deformations, especially scale changes and large illumination changes, and provides much more reliable correspondences.

Dai and Li [22] proposed a saliency detection method for multiple targets based on multi-saliency detection, aiming to solve the problem of incomplete saliency detection and unclear boundaries in infrared multi-target images with different target sizes and low signal-to-noise ratio under sky background conditions. The test results show that after the experimental analysis of infrared video photographs and the comparative analysis of Receiver Operating Characteristic (ROC) curve and Area Under the Curve (AUC) index, the infrared image saliency map generated by this method has clear target details and a good background suppression effect, and the AUC index performance is good, reaching over 99%. It effectively improves the multi-target saliency detection effect of the infrared image under the sky background and is beneficial to the subsequent detection and tracking of image targets.

Albarracín-Sánchez et al. [23] presented a practical and effective method to separate and identify Partial Discharge (PD) sources acting simultaneously in high-voltage systems under test. The effectiveness of the proposed method and the developed Matlab application for separating PD sources was demonstrated with a practical laboratory experiment, where various PD sources and pulse-type noise interferences were simultaneously measured.

Nestor et al. [24] presented a lightweight image encryption algorithm, based on chaos induction via a 5-dimensional hyper-jerk oscillator network. The superior qualities of the proposed strategy are traced to the dynamic set of keys used in the substitution process, which heralds the generation of the final ciphered image. Based on the average results obtained from the entropy analysis (7.9976), NPCR values (99.62), UACI tests (33.69) and encryption execution time for 512 × 512 images (0.1141 s), the proposed algorithm is judged to be fast and robust to differential and statistical attacks relative to similar approaches.

Yu et al. [25] proposed a fringe phase-shifting, field-based, fuzzy quotient, space-oriented, partial differential equations filtering method, to reduce the phase error caused by Gaussian noise while filtering. Experiments demonstrated that the method achieves a higher signal-to-noise ratio and lower phase error caused by noise, while also retaining more edge details.

Ricolfe-Viala and Esparza [26] presented an analysis of the distortion depth dependency in strongly distorted images. This calibration method obtains more accurate results when compared to existing calibration methods.

Meyer, Wei and Jiang [27] presented “HOMER”, a cloud-based system for video highlight generation, which enables the automated, relevant, and flexible segmentation of videos. HOMER demonstrates an improvement of up to 38% in the $F_{1}$-score from baseline, while not requiring any external hardware. The portability and scalability of HOMER through the implementation of two smartphone applications was also demonstrated. This system outperforms state-of-the-art solutions by fusing internal video content-based features with the user’s emotion data.

Sebastião et al. [28] collected environmental and physiological data from firefighters (FF) to perform a heart rate (HR) analysis according to different levels of carbon monoxide (CO) exposure during firefighting. Then, based on the collected HR and on CO occupational exposure standards (OES), they presented a classifier to identify CO exposure levels through the HR measured values. They reached an overall classification accuracy of 91.9% of CO levels, using 100 bagged classification trees. This classification can be performed in real-time through minimally invasive monitored HR.

Finally, I would like to personally thank all the authors and reviewers contributing to this Special Issue: the former for their original ideas and solutions, and the latter for their time and valuable suggestions for improvement. Their excellent work has allowed *Sensors* journal to present novel and interesting contributions in the “Data, Signal and Image Processing and Applications in Sensors” field. Thank you to all of you! 
